# Resolution of Two Sub-Populations of Conformers and Their Individual Dynamics by Time Resolved Ensemble Level FRET Measurements

**DOI:** 10.1371/journal.pone.0143732

**Published:** 2015-12-23

**Authors:** Gil Rahamim, Marina Chemerovski-Glikman, Shai Rahimipour, Dan Amir, Elisha Haas

**Affiliations:** 1 The Goodman Faculty of Life Sciences Bar Ilan University, Ramat Gan Israel 52900; 2 Department of Chemistry, Bar-Ilan University, Ramat Gan Israel 52900; University of Leeds, UNITED KINGDOM

## Abstract

Most active biopolymers are dynamic structures; thus, ensembles of such molecules should be characterized by distributions of intra- or intermolecular distances and their fast fluctuations. A method of choice to determine intramolecular distances is based on Förster resonance energy transfer (FRET) measurements. Major advances in such measurements were achieved by single molecule FRET measurements. Here, we show that by global analysis of the decay of the emission of both the donor and the acceptor it is also possible to resolve two sub-populations in a mixture of two ensembles of biopolymers by time resolved FRET (trFRET) measurements at the ensemble level. We show that two individual intramolecular distance distributions can be determined and characterized in terms of their individual means, full width at half maximum (FWHM), and two corresponding diffusion coefficients which reflect the rates of fast ns fluctuations within each sub-population. An important advantage of the ensemble level trFRET measurements is the ability to use low molecular weight small-sized probes and to determine nanosecond fluctuations of the distance between the probes. The limits of the possible resolution were first tested by simulation and then by preparation of mixtures of two model peptides. The first labeled polypeptide was a relatively rigid Pro_7_ and the second polypeptide was a flexible molecule consisting of (Gly-Ser)_7_ repeats. The end to end distance distributions and the diffusion coefficients of each peptide were determined. Global analysis of trFRET measurements of a series of mixtures of polypeptides recovered two end-to-end distance distributions and associated intramolecular diffusion coefficients, which were very close to those determined from each of the pure samples. This study is a proof of concept study demonstrating the power of ensemble level trFRET based methods in resolution of subpopulations in ensembles of flexible macromolecules.

## Introduction

The native conformations of proteins are stabilized by a complex network comprising a very large number of interactions, which leads to a narrow ensemble of conformations, as opposed to the unfolded state and partially folded state ensembles that include a large number of nonnative and highly flexible conformations [1–3]. Characterizing such ensembles requires statistical representations i.e. distributions of distances, their mean, variance, and rates of fast fluctuations. In many cases two sub-populations or more can be resolved in ensembles of flexible biopolymers [4–9]. Each sub-population of the ensemble has a characteristic distribution of intramolecular distances and rates of fast fluctuations. For example, in a two-state protein folding transition [10, 11], the sub-population of very flexible unfolded molecules folds to a less flexible native state. In this kinetic process, the distance distribution parameters of each sub-populations remain constant, while only the ratio between the two subpopulations is shifted [12].

FRET-based experiments are widely used in studying biopolymer structure and function [13–15]. Methods based on time resolved FRET measurements (trFRET) at the ensemble level are widely used to determine distributions of inter- and intra-molecular distances in biopolymers and their sub-populations [6, 12, 16, 17]. Major advances in resolution of sub-populations in ensembles of biopolymers were achieved by the application of single molecule detected FRET (smFRET). These methods are widely used to identify/characterize subpopulations in ensembles of biopolymers based on histograms of transfer efficiencies [18–23]. The strength of the relevant smFRET based methods in this context is a result of the ability to monitor each photon and burst of photons individually and “construct” the ensemble statistics by sorting large number of single photon events. Thus the statistics is analyzed by model-free procedures [24–26]. Progress in enhancement of the photon flux and photo-protection agents and application of correlation analyses constantly enhance the signal to noise ratio and the time resolution of smFRET experiments available for determination of conformational heterogeneity and dynamics [27–35] Methods for computation of the fluorescence correlation and cross correlations of photon emission times by both probes (donor and acceptor) further enhance the time resolution of smFRET measurements [36–39] [40]Advanced smFRET methods [41] were developed [42–45] e.g. methods where the lifetime of excited states of the probes can be determined. [23].

Ensemble based time resolved FRET (trFRET) experiments make use of the distance dependence of transfer efficiency and the effect of the fast fluctuations, both of which affect the fluorescence decay curves of both the donor and the acceptor [46]. Both unbiased intramolecular distance distributions at <2Å resolution and intramolecular diffusion coefficients representing fluctuations at sub-nanosecond to hundreds of nanosecond time regime can be obtained. [46, 47]. Global analysis of both the donor and the acceptor decay curves reduce the uncertainties due to the correlation between the width and the fast reconfiguration in ensembles of multiple conformers studied by trFRET measurements [48–50]. Analysis of trFRET experiments is not model free as is the case with smFRET experiments. However, the use of natural probes such as the aromatic amino acids or their analogs [51, 52] enable reduction of uncertainties related to the size of the probes currently in use in smFRET experiments [25]. trFRET experiments can be applied with a wide range of pairs of probes including pairs with R_o_ values shorter than Ca. 25Å not commonly used in smFRET experiments. Therefore there are applications where it might be of advantage to use the ensemble based trFRET methods in studies of conformational transitions and dynamics in ensembles of biopolymers. This motivated us to explore the feasibility of resolving two sub-populations in ensembles of biopolymers and resolution of different intramolecular diffusion coefficients associated with each one of the two sub-populations.

Here we present a “proof of concept” of a method for simultaneous determination of the intramolecular distance distributions in two sub-populations in an ensemble of biopolymers, together with the ratio of the two populations and the associated intramolecular diffusion coefficient of each population of conformers based on global analysis of trFRET experiments. The concept is validated by analysis of trFRET measurements of solutions of two different synthetic polypeptides mixed at different concentrations ratios. The parameters of the distance distributions of each polypeptide were recovered from the measurements of the mixtures, and were very close to the parameters obtained when each polypeptide was measured separately, in pure solutions. The recovered concentration ratios were also close to the ratios of the preparation. The limits of the method were tested both by simulation and by changing the concentration ratios in the trFRET experiments.

## Materials and Methods

### Theory

The change of the donor probe excited state in the presence of an acceptor is given by Eq (1) [50]:

$$\frac{\partial \bar{N}(r,t)}{\partial t}=-\frac{1}{\tau_{d}^{0}}\left[1+{\left(\frac{R_{0}}{r}\right)}^{6}\right]\bar{N}(r,t)+D\frac{1}{N_{0}(r)}\frac{\partial }{\partial r}\left[N_{0}(r)\frac{\partial \bar{N}(r,t)}{\partial t}\right]$$ (1)

The first term in the right-hand of the Eq 1 is the donor de-excitation by either the Förster mechanism or through spontaneous emission, and the second term is the contribution of the intra-molecular dynamics in a potential field. The population of exited donor probes at the time of excitation, *N* ^*^(*r*,*t*) at t = 0, is proportional to the equilibrium distance distribution N0(r); and $\bar{N}(r,t)=N^{*}(r,t)/N_{0}(r)$; Flory[53] pointed out that even in fully disordered states of polymers in general and polypeptides in particular, the distributions of end to end distances of short chain segments cannot be described by a perfect Gaussian function due to the length of the persistence vector and the excluded volume effect. Therefore in our analyses we use model function of skewed Gaussian form which can accommodate either perfect Gaussian shape or skewed Gaussian shape. We routinely fit the data with the two parameters skewed Gaussian expression, $N_{0}(r)=4\pi r^{2}e^{-b{\left(r-a\right)}^{2}}$ as a model for determination of the radial intra-molecular distance distribution. In this expression '*a*' and '*b*' are parameters determining the distribution’s mean and width, respectively. Introduction of a second parameter in the model distribution function increases the correlation between the parameters [54]. All attempts to fit experimental data of the flexible model peptide used in the present study, with a single parameter Gaussian function failed to yield acceptable quality of fit.

*D* is defined as the intra-molecular diffusion coefficient of the segments carrying the two probes. We ignore possible dependence of D on the distance r; *R0* is the Förster radius. The differential Eq (1) does not have an analytical solution and a numerical method is used for obtaining the decay of $\bar{N}(r,t)$ for each sub-population. In the case of analysis of trFRET of mixtures of two sub-populations, the decay curves of the reduced population size of each sub-population, $\bar{N_{1}}(r,t)$ and $\bar{N_{2}}(r,t)$, are multiplied by the corresponding equilibrium sub-population distributions at t = 0, *N*_0,1_(*r*) and *N*_0,2_(*r*), respectively, which are free parameters calculated by the curve fitting procedures along with the parameters $\tau_{d}^{0}$, the lifetime of the donor in the absence of an acceptor, and the molar fraction of the first subpopulation, *FRX*. A calculated curve depicting the decay of the donor emission is obtained by summation of the contributions of the two subpopulations Eq (2):

$$I_{d}{}_{\left(t\right)}=c\left\{\left[\int_{R_{\text{min}}}^{R_{\text{max}}}\bar{N_{1}}(r,t)N_{0,1}(r)dr\right]FRX+\left[\int_{R_{\text{min}}}^{R_{\text{max}}}\bar{N_{2}}(r,t)N_{0,2}(r)dr\right](1-FRX)\right\}$$ (2)

In Eq 2 R_min_ is the limit of close approach of the two probes and is usually 2 Å or at least 0.1R_o_ where the effect of FRET is not detectable anyway. R_max_ is the contour length of the polymer. The fluorescence lifetime of the probes in conformers with intramolecular distances larger than Ca. 2R_o_ is only insignificantly affected by excitation transfer. Thus R_max_ should be larger than 2R_o_ but the selection of the pair of probes should be done based on the expected range of intramolecular distances of each system. FRX represents the mole fraction of the first subpopulation. *C* is a proportion parameter which represents the amount of the excited molecules due to the intensity of the excitation pulse and due to the molecules’ concentration. The parameter *D*, and the parameters of the distance distributions are highly correlated and global analysis of the donor and the acceptor fluorescence decay curves may be applied in order to reduce the uncertainty range of the computed parameters. The time dependence of the excited acceptor population which is created by the transfer of excitation from the donor, the “grow-in” component, *I*_*a*(*t*)_, is given by the decay of the donor population due to the transfer given by Eqs 1 and 2 (the ‘sink’ term) Eq 3 [50]:

$$I_{a}{}_{\left(t\right)}=c\left\{\left[\int_{R_{\text{min}}}^{R_{\text{max}}}{\left(\frac{R_{0}}{r}\right)}^{6}\bar{N_{1}}(r,t)N_{0,1}(r)dr\right]FRX+\left[\int_{R_{\text{min}}}^{R_{\text{max}}}{\left(\frac{R_{0}}{r}\right)}^{6}\bar{N_{2}}(r,t)N_{0,2}(r)dr\right](1-FRX)\right\}$$ (3)

The resulting acceptor emission decay curve is then convolved with the acceptor lifetime due to spontaneous emission $\tau_{a}^{0}$ in order to obtain the decay of the population of molecules where the acceptor is excited by the transfer effect. The donor and the acceptor fluorescence lifetime, $\tau_{d}^{0}$ and $\tau_{a}^{0}$, respectively, are obtained by time resolved measurements in the absence of FRET (molecules labeled by donor only (DO experiment) and by direct excitation of the acceptor in the double labeled molecule at wavelength at which the donor has no absorption (the AO experiment)). The labeling plan should take into account the advantage of the condition $\tau_{d}^{0}\;/\;\tau_{a}^{0}>1$ and that these lifetimes should be in the time regime of the intra-molecular motions. Without any pre-knowledge, there are a total of 8 free parameters for this model *a*_1_, *b*_1_, *D*_1_ & *a*_2_, *b*_2_, *D*_2_ for the first and second sub-population, respectively, the concentration ratio, *FRX*, and the proportion parameter, *c*. direct excitation of the acceptor is also included in the model using the absorbance ratio of the two probes at the excitation wavelength of the donor. This factor is obtained independently by absorption spectroscopy of the two probes.

### Curve fitting method

The curves calculated by solving Eqs 2 and 3 are used to calculate of fluorescence decay curves by convolution with an instrument response function (IRF) as described elsewhere [50, 55–57].

The interprobe distance distribution function is obtained from global analysis of sets of four experimental fluorescence decay curves as described in detail elsewhere [48, 49, 57, 58]. To compare the calculated fluorescence decay curves with those obtained experimentally, the calculated curves *I*_*c*_ *(t)* are convolved with the experimentally measured excitation pulse. The quality of fit of the calculated *F*_*c*_ *(t)* and the corresponding experimental intensity, at each time interval (channel), *F(t)*, is evaluated using four criteria: (a) The global and local *χ* ^*2*^ values, (b) the distributions of the residuals, (c) the autocorrelation of the residuals, and (d) the error intervals of the calculated parameters [56, 57].

Beechem and Haas showed the strength of the global analysis of the fluorescence decay curves of both the donor and the acceptor[48]. The contribution of the excitation transfer to the acceptor emission is most pronounced in the “grow-in” part of the decay curve of the acceptor, which is the initial section of the curve. Hence a pair of probes where the donor’s fluorescence lifetime is significantly longer than that of the acceptor (both in the absence of FRET) enable more significant determination of the grow-in contribution to the acceptor decay. This results in improved resolution of the contribution of the intramolecular dynamics to the enhancement of the FRET effect. Here we used a donor probe with long fluorescence lifetime and this enhanced the significance of the determination of the two diffusion coefficients. The analyses were repeated with different initial guesses

#### Rigorous error analysis

The statistical significance of the value of any one of the free parameters that is determined by the curve fitting procedure was evaluated by the rigorous analysis procedure [48, 57]. In this procedure each one of the free parameters was fixed at different values, and the best fit was searched by allowing all other free parameters to change. The range of the values of the fixed parameters which could be fit within 95% certainty was thus obtained (66% certainty was used for the determination of that range for diffusion coefficients).

### Simulation

We produced simulated fluorescence decays of the donor and the acceptor under conditions of excitation transfer using Eqs 1–3, with pre-selected distribution parameters of two different distance distributions. The parameters used for the first sub-population were typical of the dynamics and variance of intramolecular distances in ordered and stable protein structures, i.e. frozen dynamics (D = 0Å^2^/ns), while the second population, representing a disordered polypeptide was assigned a large diffusion coefficient (D = 20Å^2^/ns) [59]. A decay curve of 10,000 counts at the peak was simulated with 3000 channels of 0.0122 ns/channel by convolution of the theoretical decay with a 50psec IRF. Photon counting (Gaussian) noise was added. The simulated data were analyzed using global analysis of four experiments: donor emission in the absence of acceptor (DO), acceptor emission in the absence of a donor (AO), donor emission of the donor/acceptor pair (DA), and acceptor emission of the donor/acceptor pair (DAA). The DO&AO decays were used as internal calibration references. We then performed rigorous analysis of the data, as described above.

### Peptide preparation

All chemicals and reagents were of analytical grade. Rink amide resin, 9-fluorenylmethoxycarbonyl (Fmoc)-protected amino acid derivatives, and all of other reagents for solid-phase peptide synthesis were purchased from GL Biochem (Shanghai, China). All peptides were synthesized automatically (Vantage, AAPPTec, Louisville, KY), using Rink Amide—AM resin and standard Fmoc chemistry. Sequential residue coupling was achieved by N,N,N′,N′-Tetramethyl-O-(1H-benzotriazol-1-yl)uronium hexafluorophosphate / N,N-Diisopropylethylamine (HBTU/DIEA, 5:10), while Fmoc deprotection was effected by 30% Piperidine in dimethylformamide (DMF). Following the Fmoc deprotection of the N-terminal groups, the linear peptides were cleaved from the resin using mild cleavage conditions of 1% trifluoroacetic acid (TFA) in dichloromethane (DCM), without affecting the side chain protecting groups. The solvents were then evaporated under reduced pressure and the linear protected peptides were divided for further synthesis of DO and DA derivatives. Both rigid and flexible DO peptides were acetylated at their N-terminals (to generate DO_1_ and DO_2_) using a mixture of acetic anhydride / DIEA (10:5) in DMF. For the synthesis of DA_1_ peptide, N-Dansyl-Alanine was used instead of Fmoc-Alanine for the last coupling reaction, which was carried out using a standard coupling protocol. In the case of DA_2_ peptide, however, the insertion of Dansyl dye was achieved by reacting the free N-terminal of the fully protected linear peptide with 5 eq. of Dansyl-chloride (Sigma–Aldrich, Rehovot, Israel) and 1.5 eq. of DIEA in DMF solution, overnight. The completion of acetylation and dansylation reactions was monitored by reversed phase (RP) C18 analytical HPLC and mass spectroscopy (MS). Next, all the linear peptides were fully deprotected by acidolysis (TFA:triisoprpylsilane:H2O; 95:2.5:2.5 v/v), purified using RP C18 preparative HPLC column, lyophilized, and analyzed using high-resolution MS performed on a Bruker Autoflex III MALDI-TOF/TOF mass spectrometer (Bruker, Germany) in positive ion reflector mode (Table 1).

**Table 1:** Synthesized peptides for validation of the method.

Peptide^a^	Sequence^b^	m/z (g/mol),		Retention time (min)^c^
		Calculated	Observed MH^+^	
DO_1_	Ac-Ala-(Pro)_7_-Ala-Nal-Ser-Arg-Gly-NH_2_	1377.7194	1378.7141	12.31
DA_1_	Dansyl-Ala-(Pro)_7_-Ala-Nal-Ser-Arg-Gly-NH_2_	1568.7598	1569.8499	13.38
DO_2_	Ac-Ala-(Gly-Ser)_7_- Ala-Nal-Ser-Arg-Gly-NH_2_	1706.7245	1707.7270	10.31
DA_2_	Dansyl-Ala-(Gly-Ser)_7_- Ala-Nal-Ser-Arg-Gly-NH_2_	1897.7649	1898.7631	11.46

^a^peptides labeled by donor/acceptor only (DO_i_/AO_i_) and for FRET experiments (DA_i_).

^b^ Nal—L-naphthyl-alanine

^c^ RP-C18 HPLC, GraceVydac RP-18 column (DENALI; 150 mm X 2.1 mm; 3 micron), 0.3 mL/min flow rate; 0.1% TFA in water (A); and in acetonitrile (B). Gradient: 2 min of 10% B followed by an increase to 100% B over 25 min.

### Time-Resolved FRET Measurements

The time-correlated single-photon counting method was used. The excitation source was a femtosecond Ti sapphire laser (Chameleon, Coherent). The laser output was frequency tripled by a flexible second and third harmonics generator (A.P.E). A pulse selector (A.P.E) was used to reduce the basic 80 MHz pulse rate to 4.0 MHz. The excitation was at 280nm and 320nm for the donor and the acceptor, respectively. The emission wavelength was selected by a double 1/8 m subtractive monochromator (DIGIKROM CM112, Albuquerque, NM) and directed to the surface of a fast photomultiplier (Hamamatsu, R9880U-210) biased at -1100 V. The Nnaphthyl-alanine (donor) emission was collected at 340 nm (emission bandwidth 20 nm) and the Dansyl-alanine (acceptor) emission was collected at 550nm. A single-photon counting board (SPC 630; Becker and Hickel GmbH) fed via a preamplifier (HFAC-26DB 0.1UA, Brookline MA) and triggered by a photodiode (PHD-400N) was used for data collection. The response of the system yielded a pulse of full-width at half-maximum (FWHM) of 200 ps. The system was routinely checked for linearity and time calibration by determination of the decay kinetics of anthracene in cyclohexane (Merck, NJ) (decay lifetime is 4.1 ns at 350 nm). The emission was collected with a polarizer at the magic angle (54.7°) relative to the excitation polarization. The reference excitation pulse profile used for deconvolution of the experimental decay curves was a scattered light pulse generated by placing a glass in the cell. All measurements were done at 25°C in the presence of 50mM TRIS buffer at pH = 7.5. Peptides were measured at a concentration of 10μM. Four fluorescence decay curves in each set of energy transfer experiments were measured. These were (a) the fluorescence decay curve of the donor (Naphthyl-alanine) in the absence of an acceptor for both the polyproline and poly(serin-glycine) peptides (DO_1_ and DO_2_ respectively), (b) the fluorescence decay curve of the donor residue in the presence of the acceptor (DA_1_ and DA_2_), (c) the fluorescence decay curve of the acceptor (Dansyl-alanine) in the absence of the donor (AO_1_ and AO_2_), and (d) the fluorescence decay curve of the acceptor residue in the presence of the donor (DAA_1_ and DAA_2_). The background emission was routinely subtracted from the corresponding fluorescence decay curve. To measure background emission, the buffer solution was used and photons were accumulated over period of time proportional to the duration of measurement of the corresponding decay curve. Data acquisition for each set of measurements was performed on the same day within a short time period. This reduced possible variations due to changes in calibration of instruments. Samples were routinely magnetically stirred during the measurement.

## Results

### Simulation

**Fig 1:**
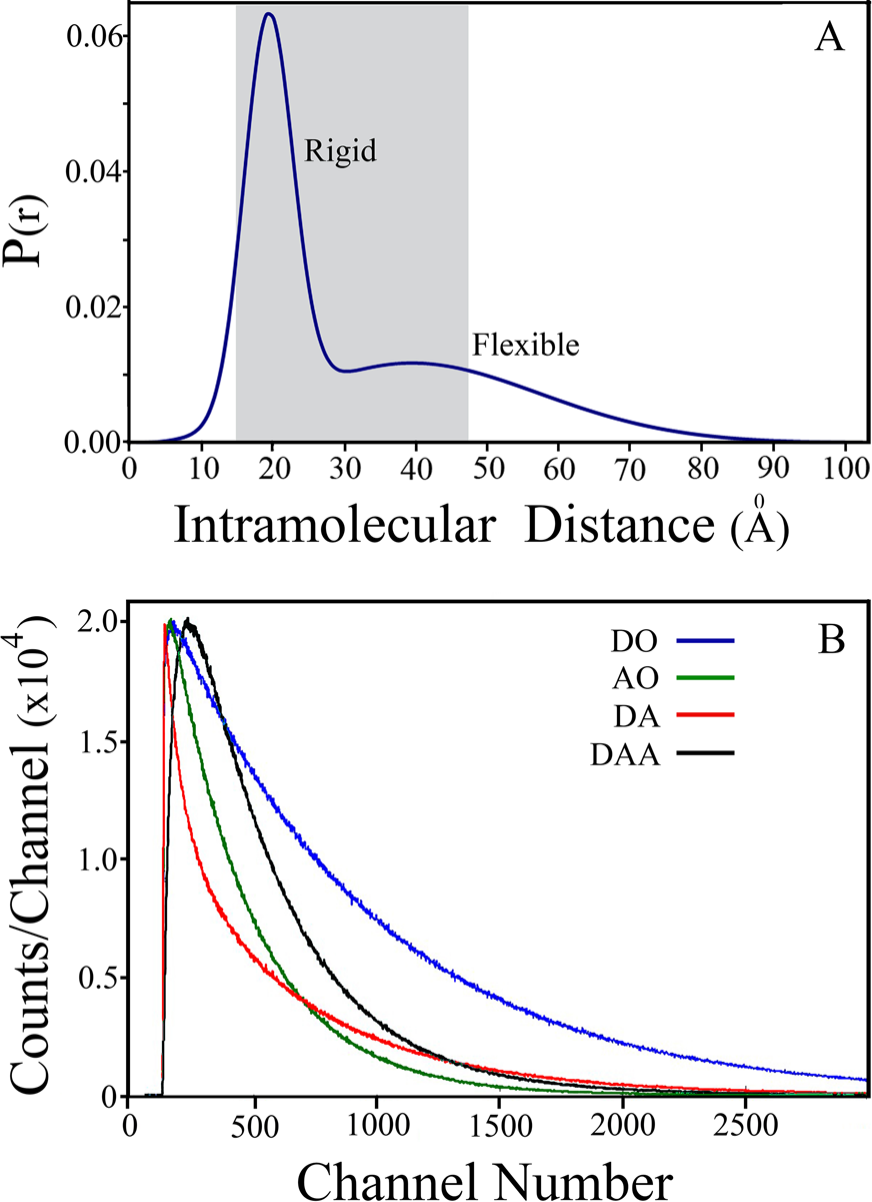
Simulated combined distance distribution of two peptides and expected trFRET data. A mixture of two peptides at a molar fraction of 0.5 each was simulated using the parameters given in Table 2 were used as input. (A) Plot of the combined end to end distance distribution expected for the ensemble formed by the mixture of the two peptides. The gray window marks the range of distances around the Förster critical distance where a significant FRET effect is expected ((0.5–1.5)R_o_). (B) Simulated fluorescence decay curves to be used in the global analysis procedure: blue (DO), fluorescence decay of the donor in the absence of acceptor; green (AO), fluorescence decay of the acceptor in the absence of a donor; red (DA), fluorescence decay of the donor in the presence of a acceptor; and black (DAA), fluorescence decay of the acceptor in the presence of the donor and under excitation at the wavelength of the donor absorption.

**Fig 2:**
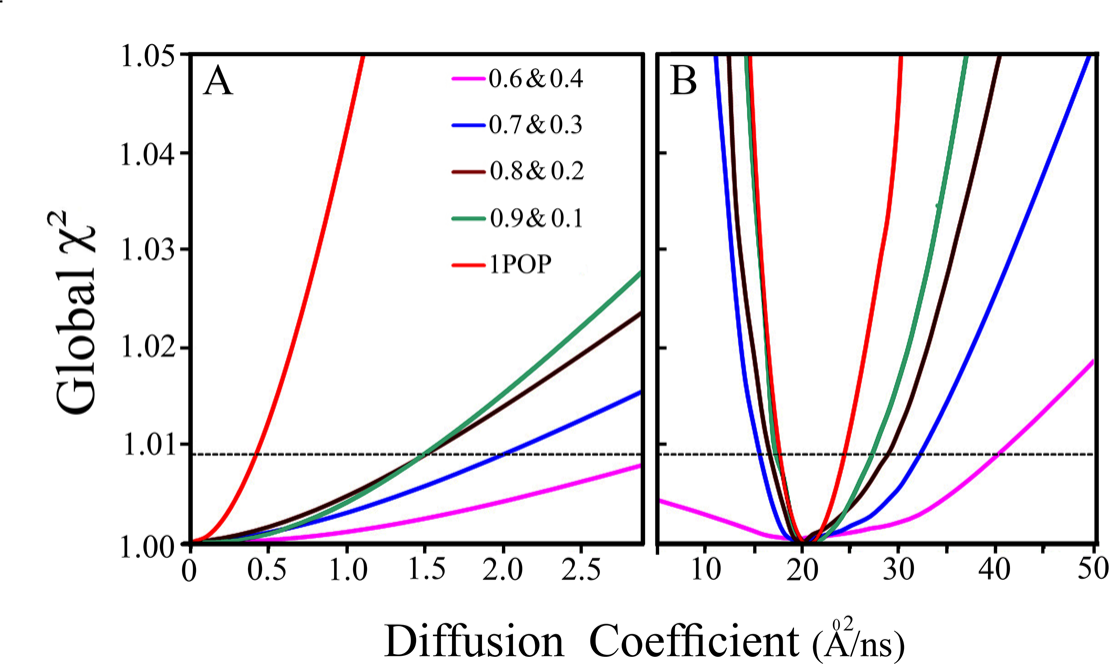
Limitations of an analysis based on single composition of the mixture of peptides. Uncertainty ranges of the two diffusion coefficients obtained by rigorous analysis of two simulated trFRET datasets. (A) The range of the value of the diffusion coefficient of the rigid peptide obtained at analysis of experiments simulated for different mole fractions of that peptide (the compositions are shown in the inset). The input parameters used for the simulations are shown in Table 1. Each trace represents the extreme values of the diffusion coefficient obtained for the indicated combinations of the molar ratios of the two sub-populations ((A) and (B) The same procedure was applied in search for the uncertainty range of the values of the diffusion coefficient for the second sub-population (D = 20Å^2^/ns). Greater reduction of the uncertainty of the two determined diffusion coefficients was obtained when experiments with low and high molar fractions of the rigid peptide 0.1 & 0.9 (red) were used. The horizontal dashed line represents 1 SD confidence level.

The expected uncertainty ranges of the values of the parameters of two distance distributions and the associated diffusion coefficients and the mole fraction of each one of the two sub-populations were studied by analysis of simulated experiments (Fig 1 and Table 2) [60]. First, we simulated and analyzed trFRET data corresponding to each sub-population separately, and recovered the parameters of each one of the synthesized distributions. The recovered values of the diffusion coefficient, with 1 SD confidence level, were D1 = 0–0.4Å^2^/ns and D2 = 18-24Å^2^/ns for the rigid and the flexible sub-population, respectively (Fig 2). The returned distance distribution parameters, with 2SD confidence level, were Mean1 = 19.3±0.2Å FWHM1 = 8.0±0.5Å and Mean2 = 39±1Å FWHM2 = 39 Å (37-46Å) for the first and the second sub-populations respectively. The next step was simulation and rigorous analysis of four fluorescence decay curves (Fig 1B) where two sub-populations were assumed with a molar ratio of 1:1 of the two subpopulations. The resolution of the two subpopulations was satisfactory. While the returned values of the first sub-population were with a narrow error range, the error range obtained for the FWHM value of the second (flexible) sub-population was too large. This phenomenon is related to the correlation between the D parameter and width parameters. Analysis of trFRET experiments of one mixture based on the donor fluorescence decay alone is limited to a few possible compositions. In contrast, joint global analysis of both probes in a series of simulated trFRET experiments of several mixture compositions of the same two subpopulations enabled recovery of the input parameters of each distance distribution with varying ranges of uncertainty. The joint analysis of multiple compositions of the mixtures reduces the uncertainty in determination of the two diffusion coefficients [58, 61]. We tested the strength of this mode of joint global analysis using synthesized decay curves of trFRET experiments with fluorescence decay at different molar ratios, and global analysis of several mixtures with different ratios of the two components. This procedure enabled reduction of the uncertainty of the analysis parameters (Fig 2).

**Table 2:** Parameters of simulated trFRET using a 2 sub-population model, each one with different intra-molecular dynamics.

peptide	Input parameters	Input value	One population model	Analysis of a simulated dataset of single composition^1a^	Joint analysis of two simulated datasets of two compositions ^1b^
Rigid	Mean^2^	19.3 Å	19.3±0.2Å	19.3±1Å	19.3±0.2Å
	FWHM^3^	8.0 Å	8.0±0.5Å	8.0 (6–15) Å	8.0±1Å
	D1*^4^	0 Å^2^/ns	0–0.4Å^2^/ns	n.s.^10^	0–1.4Å^2^/ns
Flexible	Mean^2^	39 Å	39±1Å	39±2Å	39±1Å
	FWHM^3^	39 Å	39(37–46) Å	n.s.	39(33–48) Å
	D2*^5^	20 Å^2^/ns	18-24Å^2^/ns	n.s.	18-27Å^2^/ns
	R_o_ ^6^	32 Å			
	FRX^7^		1.0(fixed)	0.5±0.15	0.1±0.03Å0.9±0.01Å
	τ^o^_d_ ^8^	10ns			
	τ^o^_a_ ^9^	4ns			

^1a^Two intramolecular distance distributions were simulated with two different sets of parameters. The corresponding fluorescence decay curves of the donor and the acceptor in the presence and in the absence of FRET were simulated assuming a 1:1 ratio of the two subpopulations. Global analysis of the donor and the acceptor fluorescence decay yielded a set of recovered parameters of the distance distributions.

^1b^Same as in 1a but two simulation experiments, assuming two different compositions of the mixtures, were jointly analyzed.

^2^Mean of the distance distribution.

^3^Full width at half maximum of each distribution.

^4,5^Intramolcular diffusion coefficient of the chain ends of the rigid and the flexible peptides, respectively.

^6^Förster critical distance.

^7^Molar fraction of the rigid peptide.

^8^Fluorescence lifetime of the donor in the absence of FRET.

^9^Fluorescence lifetime of the acceptor in the absence of FRET.

^10^Large range of uncertainty of the parameter.

The parameters recovered by joint global analysis of two mixtures (with rigid peptide fractions of 0.1 and 0.9) were D_1_ = 0–1.4Å^2^/ns, for the first sub-population (where the input was 0Å^2^/ns), and D_2_ = 18-27Å^2^/ns for the second sub-population (where the input was 20Å^2^/ns). The recovered molar ratios were 0.1±0.03Å and 0.9±0.01Å, respectively. The high uncertainty range of the recovered FWHM of the distance distribution computed for the flexible sub-population reflects the inherent correlation of this parameter with the diffusion coefficient. Additional simulations identified the experimental factors that could be modified in order to reduce the uncertainties of the recovered parameters of the distance distributions. These include the ratio of the fluorescence lifetime of the donor and the acceptor in the absence of FRET, $\tau_{d}^{0}\;/\;\tau_{a}^{0}$ and optimization of the R_o_ values such that the mean of both distance distributions is within the range of efficient transfer. The simulation study further suggested that the uncertainty ranges in determination of the distance distributions and associated diffusion coefficients is lower in cases where the FWHM of the distance distributions is lower, or when common values of intramolecular diffusion coefficients are to be found (e.g. 10Å^2^/ns as is the common case for unfolded polypeptides [62] and probably less for intermediate folding states.) Larger differences in the transfer efficiency between the two sub-populations, are associated with reduced uncertainties of the distributions’ parameters (see the discussion section). The experimental limit can change and need to be determining for each experimental setup. Prior knowledge of the parameters of one of the subpopulations (e.g. independent characterization of the native ensemble) enables further reduction of uncertainties.

### Experimental proof of concept

**Fig 3:**
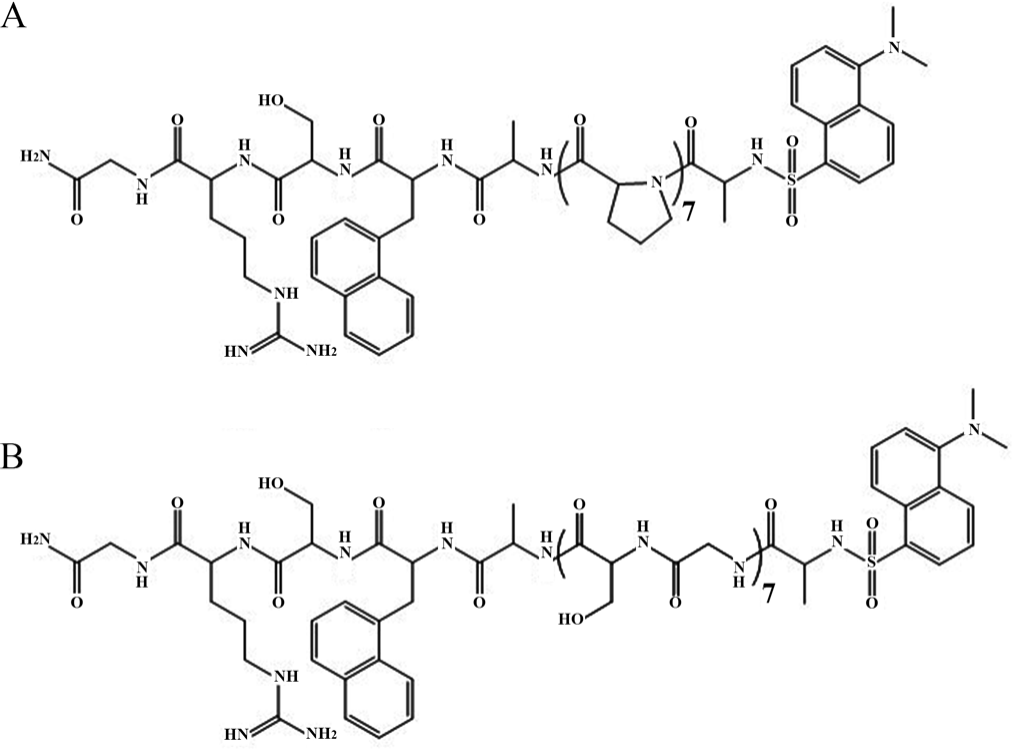
Synthetic double labeled oligo-peptides. Two oligopeptides were prepared in order to demonstrate the resolution of two sub-populations characterized by two different intramolecular diffusion coefficients in a mixed ensemble by trFRET measurements. (A) oligo-proline based rigid peptide (DA_1_). (B) oligo(Ser-Gly) based flexible peptide (DA_2_). Both peptides were labeled with naphthyl-alanine and dansyl-alanine.

The limit of resolution of determination of two different diffusion coefficients in mixed conformers was tested experimentally using two model peptides. Two peptides were synthesized, a rigid oligo-proline based peptide and a flexible oligo-(serine-glycine)_7_ (Ser-Gly) based peptide. The ends of the two peptides were labeled by the pair naphtyl-alanine (donor) and dansyl (acceptor) (Fig 3).

**Fig 4:**
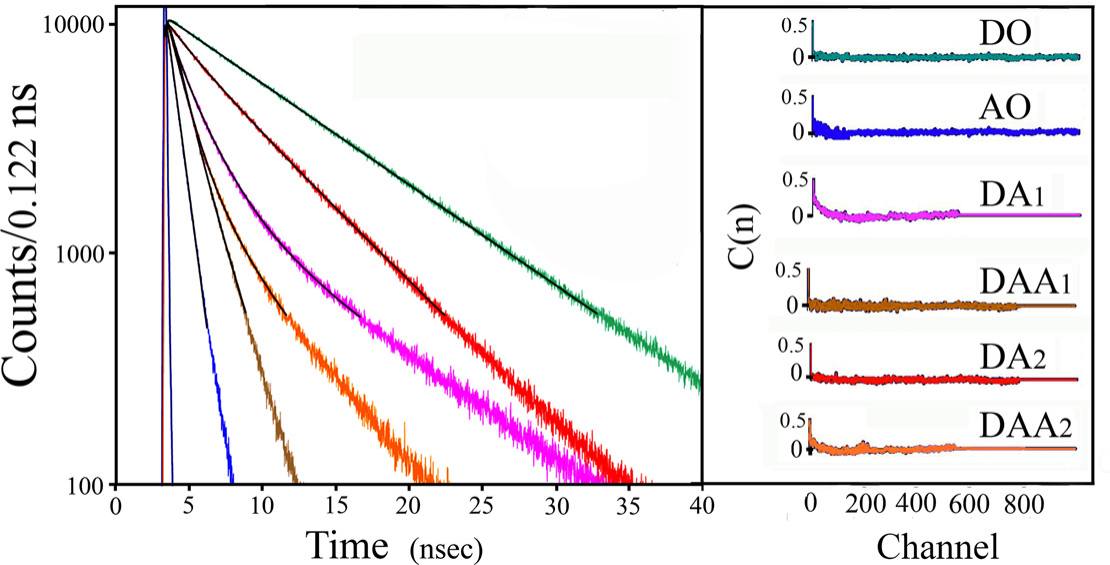
Fluorescence decay curves obtained for the two model peptides included in the global analysis. Green trace **(DO):** The donor emission decay without acceptor; the same traces were obtained for the flexible and the rigid peptide. Blue trace (**AO)** the acceptor emission decay in the absence of the donor; the same traces were obtained for the two **AO** model peptides. Purple trace (**DA**_**1**_ **)** the time resolved donor emission in the flexible peptide in the presence of acceptor. Red trace **(DA**_**2**_ **)** the time resolved donor emission in the rigid peptide in the presence of acceptor. Light brown trace **(DAA**_**1**_ **)** the acceptor emission in the flexible peptide in the presence of a donor under excitation at the wavelength of the donor absorption. Orange. Trace **(DAA**_**2**_ **)** the acceptor emission in the rigid peptide in the presence of a donor excited at the donor absorption wavelength. The black traces are the calculated theoretical curves of the best fit. Upper right inset: The right hand box: The autocorrelation of the residuals between each one of the above experimental emission decay curve and the corresponding best fit theoretical emission decay curves (black traces) obtained by the global analysis.

**Fig 5:**
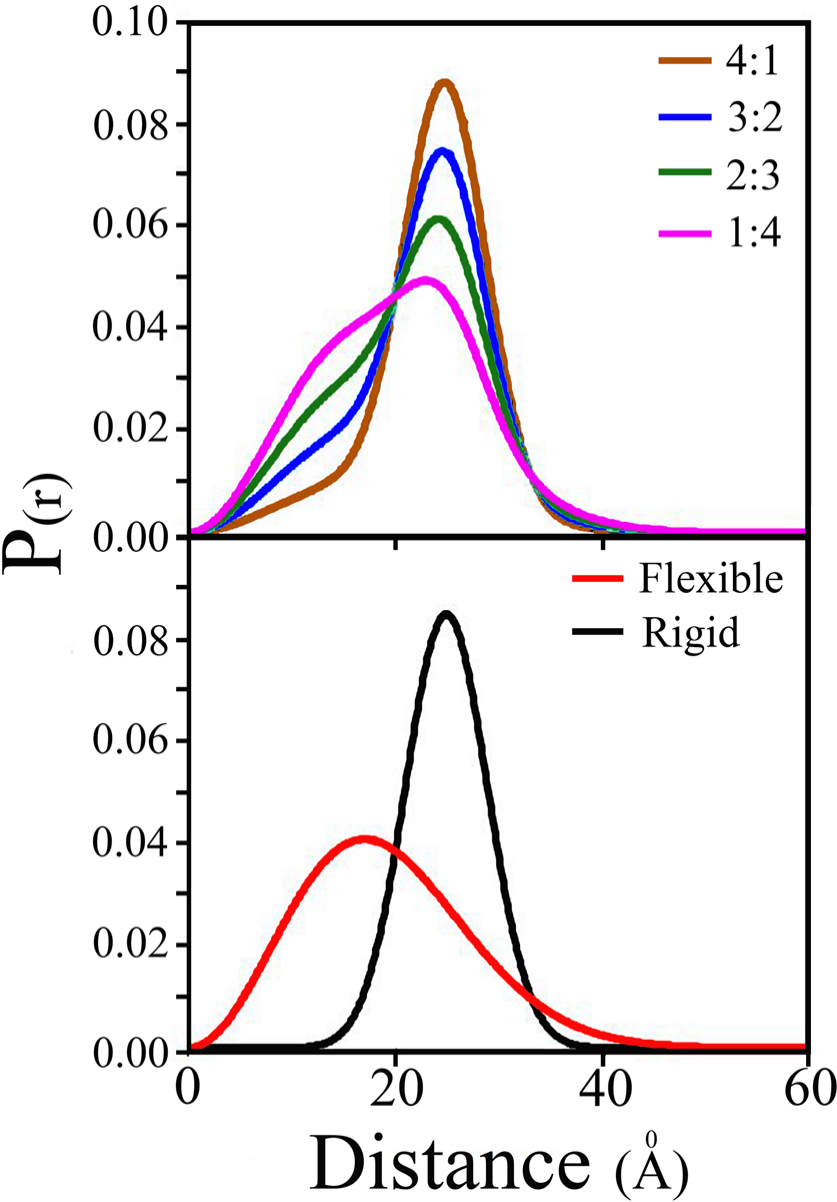
End-to-end distance distributions obtained for the mixtures of the model peptides by the joint global analysis. Results of joint analysis of trFRET data given in Table 3. Top panel: sum of 2 sub-populations with different rigid:flexible mixture ratios: brown 4:1, light blue 3:2, green 2:3, magenta 1:4. Bottom panel: Separate measurement of single population distance distributions of the flexible peptide (red) and the rigid peptide (black). The recovered single population parameters from the top panel were equal to those obtained by the separate measurement of each one of the peptides as shown at the bottom panel.

trFRET measurements were applied to each one of the two double labeled (DA) peptides and corresponding donor only (DO) labeled peptides (Fig 4). Joint analysis of these experiments yielded the end-to-end distance distribution and the intra-molecular diffusion coefficient parameters characterizing the ensemble of each of the two peptides in solution (Table 3, Fig 5). Mixtures of the two DA labeled peptides were prepared at known molar ratios, the fluorescence decay of both the donor and the acceptor were measured, and the global analysis algorithm applied assuming the two subpopulation model (Table 3, Fig 5). Both analyses were conducted using a Förster radius (R_o_) of 22Å [63, 64]. In these analyses, the excited state lifetimes of the two probes in the absence of FRET were also free parameters, and were recovered by the joint global analysis of the series of decay curves. The resulting mean fluorescence lifetimes of the donor and of the acceptor were 35.5±0.2ns and 3.3±0.1ns, respectively, for the flexible peptide, and 34.9±0.2ns and 3.8±0.1ns, for the rigid one. These lifetimes served as an internal reference in the global analysis procedure (see Methods). In this analysis, the ratio of the two sub-populations was a free parameter, and as can be seen in Table 3, the preset composition of the mixture was successfully recovered by the analysis. Comparison of the parameters of the end to end distance distributions obtained for each one of the two model peptides when measured in pure solution and those obtained from global analysis of the of the trFRET measurements of the mixtures of these peptides shows successful determination of two different distributions of intramolecular distances and the associated individual diffusion coefficients. We conclude that by using this method we can determine simultaneously and without any prior knowledge, both the distance distribution and diffusion coefficients of sub-populations in ensembles of mixed conformers by means of trFRET measurements and global analysis.

**Table 3:** Parameters of the distributions of end to end distances of model peptides obtained by global analysis of trFRET measurements applied to preparations of the two model peptides in pure solutions, and in a series of mixtures of different ratios.

Model	Parameters obtained ^a^	Pure solutions ^b^	χ^2^ ^c^	Mixtures of the two model peptides ^d^	χ^2^ ^e^
Flexible Peptide ^f^	Diffusion coefficient (Å^2^/ns)	14.2 (10.7–15.2)	1.15	12.5 (5.5–14.0)	1.13
Flexible Peptide ^f^	Mean (Å)	17.5 (17.3–17.5)	1.15	17.0 (16.5–17.5)	1.13
Flexible Peptide ^f^	FWHM (Å)	20.3 (15.1–20.3)	1.15	19.4 (13–20)	1.13
Rigid Peptide ^f^	Diffusion coefficient (Å^2^/ns)	0.2 (0.2–0.3)	1.08	0.2 (0.0–0.6)	1.13
Rigid Peptide ^f^	Mean (Å)	25 (24.5–25.5)	1.08	24.8 (23.0–25.5)	1.13
Rigid Peptide ^f^	FWHM (Å)	12 (9.9–13.8)	1.08	9.0 (6.0–15.0)	1.13
Fraction ^g^	0.8			0.81 (0.75–0.83)	1.13
Fraction ^g^	0.6			0.59 (0.47–0.63)	1.13
Fraction ^g^	0.4			0.40 (0.22–0.46)	1.13
Fraction ^g^	0.2			0.22 (0.00–0.30)	1.13

^a^The parameters of each distribution, which were determined by the joint global analysis of the donor and the acceptor fluorescence decay.

^b^Values of each parameter obtained by analysis of trFRET experiments of a separate solution of each peptide.

^c^Best fit χ^2^ values obtained in the analysis of trFRET experiments of each peptide in pure solution.

^d^Values of the parameters of the end to end distance distribution and intramolecular diffusion coefficients of each subpopulation obtained by joint global analysis of trFRET, monitored in a series of mixtures (four compositions),

^e^Global χ^2^ of the joint analysis.

^f^Separate measurements of the two peptides.

^g^The series of compositions of the mixtures of the model peptides included in the joint global analysis of the multiple trFRET experiments, the fraction of rigid peptide is shown

## Discussion

Determination of fast dynamics of macromolecules in ensembles of more than one conformational state is an important challenge in current molecular biophysics. The fast advancement of smFRET based methods makes such methods the first choice in studies of ensembles of biopolymers of mixed populations of conformers. Yet, in some applications the trFRET based method presented here may be the method of choice and hence here we report a proof of concept of the method. We demonstrate that by combination of ensemble trFRET measurements and joint global analysis it is possible to simultaneously determine both the means and widths of the distributions of intramolecular distance and diffusion coefficients in two different sub-populations in ensembles of biopolymers. We show that this can be achieved in the absence of any auxiliary source of data.

### Limits of the achievable resolution

The limits of resolution of the parameters (diffusion and the parameters of the distance distribution) by the method presented here depend on the combination of all characteristics of the system studied. Therefore a general set of limits of resolution can be obtained only by an extremely large number of simulations and is not practical. Yet, some indications of the limits of resolution can be outlined. For each dataset the limits of resolution are routinely estimated for each parameter by the rigorous analysis procedure [55] where each parameter is varied step by step, the other parameters allowed to change in search for the best fit. The limits obtained by such analyses are shown in Tables 2 and 3 (in the parentheses). Some additional test cases are shown in Table 2. The input of the simulation presented in Table 2 is an example of two sub-populations of a protein, the folded (rigid) state and the unfolded (flexible) state. The larger the differences in the transfer efficiency between the two sub-populations, the lower are the uncertainties of the parameters. The smaller the values of the FWHM the lower are the uncertainties in the values of the parameters. The simulation used in Table 2 where one subpopulation has large FWHM (e.g. both mean and width are 39Å as is the common case for unfolded polypeptides) is a case of lower limit of resolution which is shown as uncertainties of the parameters. In this case the limit depends on the difference in the integrated transfer efficiency of the two subpopulations. A minimum of 25% difference in the transfer efficiency between the folded and the unfolded sub-populations is needed in order to achieve the resolution of the diffusion parameter in that simulation.

Additional simulations show that the lower the value of the diffusion coefficients, the lower the uncertainties. The value of the input diffusion coefficient used in the simulation of the flexible (unfolded) sub-population in the parameters set presented in Table 2 (20 Å^2^/ns) is close to the upper limit of the diffusion coefficient found in disordered polypeptides[59, 65]. Thus the resolution obtained with this set of parameters is a lower limit for practical cases and is enhanced the smaller the value of the diffusion parameter. The limit of resolution of the size of the two subpopulations and its effect on the uncertainty of the determination of the value of the diffusion parameter is shown in Table 2 and Fig 2. As shown in Table 2, analysis of a single experiment where only one mixture of two subpopulations at a ratio of 1:1 (FRX = 0.5) cannot recover the value of the diffusion coefficient with any acceptable limits of uncertainty. But joint analysis of two simulated compositions (mixture ratios more than 0.4&0.6 (Fig 2 and Table 2)) results with acceptable resolution of the diffusion parameter and the parameters of the distributions.

The Förster critical distance, *R*_*o*_, is critical factor in setting the limits of resolution of the parameters. Maximal resolution is achievable when the means of both distributions of the intramolecular distance are within the range of (1±0.5)R_o_ (Fig 1). For the input parameters in Table 2, the limits of R_o_ values for acceptable resolution of the parameters are 26Å< R_o_ <37Å.

In each trFRET experiment selection of the sites of labeling of a macromolecule (control of the measured mean distances) and the selection of the pair of probes (for optimized *R*_*o*_ value) are in the hands of the investigators and allow for optimization of the system for improved resolution.

### Global analysis of conformational transition

The uncertainty of the parameters obtained by this method was reduced by the joint global analysis of both the donor and the acceptor in a series of mixtures. This is the case in ensembles undergoing conformational transitions such as protein folding or complex formation. The fast data collection that can be achieved by the ensemble trFRET methods enable monitoring the dynamics in two time regimes. First the fast ns fluctuations (the intramolecular diffusion coefficients) and second, slower kinetics of change of parameters characterizing the subpopulations (molar ratio of the two sub-populations, mean and width of a distribution) determined by fast data collection at series of consecutive time intervals). This can be achieved by data collection from the ensemble at multiple time intervals following fast change of the solution conditions. A typical case where these two time regimes are of interest is the case of protein folding transition studied by the “double kinetics” method [1, 16, 17, 66–70]. The folding/unfolding transition can be initiated either by fast mixing (stopped flow or by continuous flow[71, 72]). Another case of potential interest can be analyses of experiments in which the folding transition is studied under equilibrium in a series of solutions of increasing denaturant concentrations [12, 26]. Donor probes with long fluorescence lifetime (10–100 ns) are available and can be effective in studies of the internal friction[32, 34, 46] of protein molecules in the unfolded and partially folded states and its effect on the folding transition [73] [62, 74, 75]. The ensemble level trFRET measurements can utilize natural probes (e.g. tryptophan residues) or non-native amino acids (cyano-phenylalanine,[76] naphthyl-alanine etc.). Site specific substitution by such probes minimizes the chance for structural perturbations. Small size probes suitable for site specific attachment to protein backbone (e.g. via alkylation of cysteine residues) are available and also contribute to reduction of possible structural perturbation. The procedures for preparation of homogenous samples of single and double labeled protein molecules are thus simplified. The available arsenal of probes enables easier optimization of design of experiments where distances in the range of 10 to 30Å are of interest. This is particularly powerful when studies of specific conformational transitions at the sub-domain level (e.g. helix-coil transition of a specific helix in a protein molecule) are of interest [77, 78]. Moreover, the smaller size of the probes reduces the probes’ contributions to the measured distances and their fluctuations. Ideal probes are those that exhibit mono-exponential decay of excited states. However, methods of analysis were developed, and it was shown that even for pairs of probes that have multi-exponential rates of decay of the excited state, the parameters of the distance distributions can be resolved with an acceptable range of uncertainty [57, 79].

Analysis of ensemble level trFRET measurements can be done using different models based on physical parameters or in model free mode (e.g. maximum entropy analysis). In the test case presented here we could not fit the data using Gaussian distribution, the quality of fit was poor and therefore a model of two free parameters was used. The choice of the model depends on the physical question of interest. Models based on theoretical parameters can be inserted in curve fitting procedure but in the present case, as well as in protein folding studies, our goal is to resolve the sub-populations, their molar ratio and mean and width of the distance distribution. It should be born in mind that the dependence of the probability of excitation transfer on the inter-probes’ distance at the short and long ends of the distributions is weak. Therefor one should be careful in assigning physical interpretation of parameters based on specific models. Moreover, trFRET based methods can yield only the minimal number of sub-populations and there is no way to exclude the possibility of higher number of sub-populations. In the absence of physical reason for the use of two sub-population model, the standard procedure of search for improvement in the quality of fit obtained by proceeding from one population model to a two subpopulations model[57, 80].

Different conformers composing a conformational sub-population can have very different rates of fluctuations of the distance between the labeled sites. But, the method presented here resolves a single average value of the diffusion coefficient for each sub-population. The current method cannot yield higher resolution unless the distance dependence of the diffusion coefficient is known (or assumed) a priori. However, the simplicity of the preparative procedures makes the preparation of series protein samples where selected sub-domain elements are site specifically labeled and individual diffusion coefficients can thus be resolved [59].

New methods of analysis are emerging where molecular dynamics is used for directly simulating the decay curves of the probes measured in trFRET experiments[25, 81–83]. These methods can, and will probably enable, more detailed physical interpretation of the data, but only within the limits of uncertainty made possible by the quality of fit of the experimental data.

The effectiveness of application of the trFRET based method presented here is optimized by proper design of each experiment. Such design should include selection of probes with optimal R0, to cover the expected distance distributions and to suit the use of global analysis of the donor and the acceptor decay curves. In principle, repeated experiments using a series of preparations labeled by pairs of probes of different R0 values can further reduce the uncertainty problem rather than using the acceptor data (due to the problem of the direct excitation of the acceptor [54]). However such a method requires major preparative work and may introduce additional structural perturbations. By using pairs of probes where the absorbance of the acceptor at the wavelength of excitation of the donor is low (lower than 0.5 that of the donor) it is possible to enhance the analysis by reduction of the relative contribution of the acceptor’s emission resulting from direct excitation. A potential source of systematic errors that should be considered is the question of whether the spectral characteristics of the probes are affected by the conformational differences between the two subpopulations. An essential routine control of ensemble trFRET is the measurement of fast rotational diffusion of the probes (the naphthyl-alanine donor has zero anisotropy).

Satisfactory reduced uncertainty of all the parameters of two distance distributions and two diffusion coefficients by the method presented here is possible only by joint global analysis of a number of molar ratios, and using both donor and acceptor decay data. The method does not require the pre knowledge of the mixture ratio. However, when the parameters of the distance distributions of one of the sub-populations are known from independent experiments, it is possible to resolve the remaining parameters by analysis of a single mixture.

the mean and width of the end to end distance distribution obtained for the seven repeats of the pair Gly-Ser 17.5 (17.3–17.5)Å and 20.3 (15.1–20.3) respectively is close to the value reported by Möglich et al. [84] (38±1Å) obtained for sixteen repeats of the same pair by trFRET experiments. Yet, the diffusion coefficient found in the present study is considerably smaller than 49±2 x 10^−7^ cm^2^/sec obtained by Möglich et al. This difference can be attributed to the larger number of peptide bonds in the model studied by Möglich et al. (more than twice) which enable faster fluctuations with the same internal friction[46].

The end-to-end distance distribution of poly-proline model peptides was investigated by several researchers in recent years using mainly single molecule measurements[76, 85–87]. Comparison of the reported results is not straightforward since the size of the probes is very different, the extensions by non-proline residues are different and the methods of analysis are different. Here we obtained a mean distance of 25Å for the Pro_7_ peptide extended by two Ala residues. This is not far from ~30Å obtained by simulation for Pro_10_ peptide reported by Schuler et al. [18]. A range of minimal distances (depending on the probes) was obtained by Doose et al.[87] and our result compare with the high end of the data reported there. The end-to-end distance distribution reported by Watkins et al.[85] for a peptide of eight proline residues and three extensions (~30Å) is also close to the results of our study. The mean end to end distance that we have determined for the Pro_7_ peptide is close to the theoretical value expected for poly-proline II of that length (21.7Å)[88] if we assume that only additional 3Å are contributed by the two Ala residues and the probes.
